# Method for Targeted Cellular Seeding of Tubular Tissue-Engineered Scaffolds for Tracheal Regeneration Approaches

**DOI:** 10.1021/acsbiomaterials.5c00365

**Published:** 2025-08-07

**Authors:** Luis Soriano, Mark Lemoine, Brenton Cavanagh, Anna Johnston, Tehreem Khalid, Fergal J. O’Brien, Cian O’Leary, Sally-Ann Cryan

**Affiliations:** † School of Pharmacy and Biomolecular Sciences, RCSI University of Medicine and Health Sciences, Dublin, Ireland D02 YN77; ‡ Department of Anatomy & Regenerative Medicine, Tissue Engineering Research Group (TERG), RCSI University of Medicine and Health Sciences, Dublin, Ireland D02 YN77; § CÚRAM, Research Ireland Centre for Medical Devices, University of Galway, Galway, Ireland H91 W2TY; ∥ Research Ireland Advanced Materials and Bioengineering Research (AMBER) Centre, RCSI University of Medicine and Health Sciences, Dublin, Ireland D02 YN77; ∇ Trinity College Dublin, Dublin, Ireland D02 R590; ⊥ Cellular and Molecular Imaging Core, RCSI, Dublin 2, Ireland D02 YN77; # Trinity Centre for Biomedical Engineering, Trinity College Dublin, Dublin, Ireland D02 R590

**Keywords:** 3D printing, tracheal tissue engineering, biomaterial characterization, spatial cell seeding, collagen-hyaluronic acid scaffolds

## Abstract

Effective tracheal tissue engineering benefits from scaffolds that mimic the native structure of the tissue, provide mechanical stability, and support spatially controlled cell seeding to encourage tissue regeneration. This study presents a novel approach for fabricating tubular scaffolds for tracheal regeneration that integrates a 3D-printed polycaprolactone (PCL) backbone with a freeze-dried collagen-hyaluronic acid (CHyA) layer. Two scaffold geometries (tubular and c-shaped) were produced and mechanically characterized, and it was demonstrated that PCL reinforcement significantly enhanced scaffold structural robustness and durability. To achieve spatially selective cell seeding, custom-designed PLA accessories facilitated the precise deposition of respiratory epithelial cells (Calu-3) onto the inner layer and lung-derived fibroblasts (Wi38) onto the outer layer of the scaffolds. Monoculture experiments showed successful cell localization, while sequential seeding established an effective coculture system with enhanced epithelial coverage and sustained fibroblast viability. This study validates a scalable and customizable method for manufacturing mechanically robust tubular scaffolds with precise spatial cell organization, providing a promising platform for tracheal tissue engineering and potentially other tubular applications such as vascular or gastrointestinal regeneration. Future work will focus on validating this method with primary human cells, incorporating air–liquid interface cultures to enhance epithelial differentiation, and scaling up the constructs to anatomically relevant sizes to advance clinical translation.

## Graphical abstract

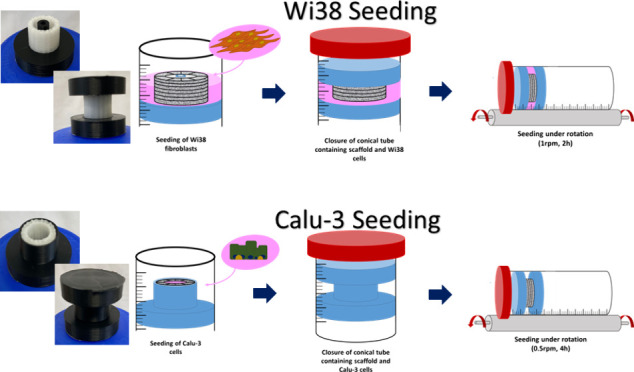

## 1 Introduction

Tissue engineering represents a growing cross-functional and multidisciplinary field where cells, biomaterials, biochemical cues and bioreactors are combined to provide tools for human tissue regeneration.^1^ Scaffolds, a main pillar in tissue engineering, are designed to provide 3D environments which mimic the tissue’s native extracellular matrix to allow for the growth, proliferation and differentiation of specific cell types.^2^ In particular, tubular scaffolds have recently gained attention for their application in the regeneration of tissue characterized by tubular geometry such as vasculature, trachea and urethra.^3–6^ Tubular scaffolds have been fabricated using a wide range of materials including natural polymers, such as collagen and fibrin, and synthetic polymers like polycaprolactone (PCL) and polylactic acid.^7–9^ For the development of such tubular scaffolds, there are certain requirements that the scaffold should fulfill. The scaffold should present the right surface and biochemical cues to support the adhesion of cells while having the desired porosity and pore size to drive cellular proliferation, differentiation and access for nutrients.^10–12^

To date, several techniques have been investigated for the production of tube-like scaffolds including electrospinning, freeze-drying, 3D printing (3DP) and casting.^5^ Freeze-drying is a widespread technique for the development of porous scaffolds as it allows the formation of a 3D porous network by the removal of a solvent present in a diluted sample containing the biomaterials that has been previously frozen.^13,14^ On the other hand, 3D printing has gained popularity for the manufacture of tissue-engineered scaffolds as it allows precise control of the scaffold architecture, dimensions and mechanical properties, meaning that scaffolds can be customized for specific patient anatomy and requirements.^15–17^ This personalization is no longer theoretical; implantable 3D printed devices have successfully been used in clinical applications. For example, bioresorbable PCL tracheal splints custom-designed from infant airway scans have been used to treat severe tracheobronchomalacia.^18–20^

When preimplantation cell seeding is required, tubular scaffolds pose a unique challenge in comparison to flat or cylindrical scaffolds, particularly when targeted seeding is the goal. Efficient cell seeding, i.e., obtaining the desired seeding density while maintaining cell viability, is critical to the success of tubular scaffolds.^21^

Tracheal tissue engineering (TET) represents an area where the effective application of tubular scaffolds could greatly increase the clinical translation of regenerative approaches for treatment of tracheal damage.^5,22^ Tracheal damage in adults is associated with traumatic injury, cancer, and tracheobronchiomalacia. In infants, congenital abnormalities such as tracheal agenesis and esophageal atresia are the leading cause for tracheal abnormalities.^22–25^ Several recent attempts have used biocompatible tissue engineered implants aimed at trachea restoration but inadequate re-epithelialization, poor mechanical properties and insufficient vascularization have hampered progress of the technology to-date.^5^ Therefore, few examples can be found in the literature where tissue-engineered trachea has been brought forward for clinical trials. One example is the use of Marlex prostheses covered in collagen, which was further enriched with growth factors and was successfully implanted into several patients.^26–28^ Decellularization has also been investigated as an approach for trachea regeneration, with decellularized cadaveric tracheas implanted in human patients. Unsuccessful results of this approach were described, mainly due to necrotic tissues and poor vascularization, highlighting the need for preseeding strategies prior to implantation as well as the importance of a prevasculature formed in the implant in order to guarantee successful implantation.^29,30^ Finally, recent reports have described the use of 3DP scaffolds for tracheal applications. For example, 3DP splints have been investigated for treatment of severe cases of tracheobronchomalacia (TBM) and in pediatric patients presenting tracheal abnormalities.^18–20,31^ Although overall little success has been achieved in the translation of tracheal tissue-engineered approaches to the clinic, these studies have highlighted the key unmet need of tissue-engineered tracheal scaffolds: provision of desired mechanical properties, adequate re-epithelization of the scaffolds and sufficient vascularization to support tissue growth and avoid necrosis.^5^

Tissue-engineered tubular scaffolds for tracheal regeneration present a unique challenge for cell seeding. Any tubular scaffold is first designed to mimic the hollow tube structure of the tracheal anatomy and then recapitulate the different layers of the trachea and its biomechanics.^22,32–34^ Ideally, preseeding a tracheal scaffold would involve respiratory epithelial cells in the inner lumen to mimic the respiratory epithelium, chondrocytes to promote cartilage formation, and endothelial cells to encourage vascularization and enhance integration with host tissue. Lung-derived fibroblasts, which contribute to the subepithelial matrix, could also support the scaffold’s integrity. These cell types collectively support full grafting by addressing functional, mechanical, and biological needs. However, achieving selective cell seeding for each layer remains a significant unmet need, as conventional methods often result in poor spatial control and limited cell viability. Therefore, any tubular scaffold for tracheal tissue engineering should encourage the growth of a respiratory epithelium in the inner lumen, while facilitating the growth of supporting tracheal tissues such as vasculature networks, mesenchymal tissues and cartilage in the outer layer(s) of the scaffold.^5^

However, a key aspect that hinders the development of these complex seeded scaffolds in the early stages is how to selectively seed different cell types in the respective layers of the scaffold. This is critical, first to mimic the tracheal tissues and second to allow for successful implantation once the scaffold had been seeded prior to implantation. One example of such an approach was reported in 2018, where mesenchymal stromal cells seeded on the luminal hydrogel-based layer of a 3DP tubular scaffold were used to demonstrate complete re-epithelialization on a rabbit model. However, this approach for targeted cell seeding into specific scaffold layers was restricted by the use of hydrogels, which encapsulate cells within the hydrogel phase of the construct.^35^ In another attempt, Lee et al. developed a bilayered 3D tubular scaffold combining electrospinning with 3D printing, with epithelial cells seeded on the inner electrospun layer and chondrocytes on the outer 3D printed layer. To ensure spatial seeding, they used a sequential seeding strategy. Epithelial cells were seeded onto the inner electrospun layer first, allowing the membrane for attachment and proliferation, while the outer layer was shielded to prevent cross contamination. Subsequently, the outer layer was seeded with chondrocytes using the scaffolds bilayered design to maintain separation. The spatial localization of the two cell types was confirmed via microscopic and histological analyses, which showed that epithelial cells adhered to the inner electrospun layer, while chondrocytes adhered to the outer 3D printed layer. Although *in vivo* evaluation demonstrated successful tracheal regeneration, how the cell selectivity was achieved in each layer of the scaffold was not clearly demonstrated.^36^

Herein, we describe an innovative methodology to allow the selective cellular seeding of different layers of a tubular scaffold for tracheal tissue engineering. Based on previously used collagen-based scaffolds for airway tissue engineering^13^ in combination with our recently described 3DP scaffold for tracheal tissue engineering,^37^ we developed a collagen-hyaluronic acid scaffold reinforced with a PCL 37 kDa 3D printed backbone. Cells were seeded onto the scaffolds using custom-made 3D printed chambers that allow targeted seeding of the different layers of the scaffold: the inner layer (IL) was seeded using respiratory epithelial cells to mimic the tracheobronchial respiratory epithelium, while the outer layer (OL) was seeded using lung-derived fibroblast as an example of cells present in the subepithelial tracheal tissues.

The motivation behind this study lies in the significant scientific and clinical challenge of engineering functional tracheal grafts that recapitulate the complex layered architecture of the native airway. Despite advances in scaffold fabrication, the ability to localize different cell types within specific regions of a 3D scaffold remains a bottleneck in the path to achieving successful *in vivo* integration. By the introduction of a modular, targeted seeding approach, the method described here addresses this limitation. This strategy could also be adapted for additional cell types to further enable the tissue engineering of functional tracheal tissue. Furthermore, the custom seeding accessories could be scaled or modified for other tubular tissues, such as vasculature, broadening the impact of this technique.

## 2 Materials

### 2.1 Reagents

Unless otherwise specified in the text, all materials and reagents were supplied by Sigma-Aldrich (Arklow, Ireland).

#### 2.1.1 Scaffold Manufacture

- Type I bovine tendon collagen (Collagen Solutions, UK).
- Hyaluronic acid sodium salt.
- Acetic acid (Fisher Scientific, Ireland).
- 1-ethyl-3-(3-(dimethylamino)­propyl)­carbodiimide (EDAC).
- *N*-Hydroxysuccinimide (NHS).
- Dulbecco’s phosphate buffer solution (DPBS).
- PCL 37 kDa pellets (VWR, Ireland))
- 99+% ACROS Organics Chloroform (Fisher Scientific).
- Sodium Hydroxide (Fisher Scientific).
- Ethanol
- Distilled water
- DI Water

#### 2.1.2 Scaffold Characterization

- JB-4 embedding kit (Polysciences, Germany)
- Neutral buffered formalin solution

#### 2.1.3 Cell Culture

- Calu-3 cell line (HTB-55, ATCC, UK).
- Wi38 cell line (CCL-75, ATCC).
- Dulbecco′s Modified Eagle′s Medium/Nutrient Mixture F-12 Ham (D8062)
- Minimum Essential Medium Eagle (M2279)
- Penicillin/streptomycin
- l-Glutamine
- Sodium Pyruvate
- 0.25% Trypsin-EDTA Solution
- Foetal Bovine Serum (FBS) (Biosera, Ringmer, United Kingdom)
- 50 and 15 mL Corning centrifuge tubes
- 6, 12, and 24 well plates
- T175 tissue culture flasks (Sarstedt, United Kingdom)

#### 2.1.4 Assays for Cellular Validation

- AlamarBlue Cell Viability Reagent (Biosciences, Ireland)
- PicoGreen dsDNA Kit
- Phalloidin–Tetramethylrhodamine B isothiocyanate
- 4′,6-Diamidino-2-phenylindole (DAPI) readymade solution
- DPBS
- Neutral buffered formalin solution
- Triton X-100
- Na_2_CO_3_
- Bovine serum albumin (BSA)
- Invitrogen ProLong Glass Antifade Mountant (BioSciences)
- Clear nail polish (Boots, Ireland).

### 2.2 Equipment

#### 2.2.1 Scaffold Manufacture

- Ultra Turrax T18 Overhead blender (IKA Works Inc., Wilmington, USA).
- Degasser (pump)
- 6 × 6 cm^2^ polytetrafluoroethylene (PTFE) plate (Mechanical Workshop, Trinity College Dublin, Ireland).
- Freeze drier
- Biobot1 3D printer (Allevi, USA).
- 0.26 mm, Micron-S Fisnar 25G metal needle (ECT Adhesive & Dispensing Solutions, Dublin, Ireland)
- Customised stainless-metal mold for tubular scaffolds (Mechanical Workshop, Trinity College Dublin).
- Airflow hood
- Ethylene Oxide sterilization system (Anprolene AN74i, Steris)

#### 2.2.2 Scaffold Characterization

- Z050 mechanical testing machine (Zwick-Roell, Germany).
- RM2255 automated microtome (Leica, Ireland).
- Tescan Mira XMU scanning electron microscope (Advanced Microscopy Laboratory, Trinity College Dublin).
- Nikon Eclipse 90i microscope (Nikon, Japan).
- Matlab software for pore size analysis

#### 2.2.3 Cell Culture

- Incubator
- Cell Hood
- Water bath
- 3D Printer
- PLA Filaments
- Falcon Tubes
- Black Rings
- Roller
- Tweezers

#### 2.2.4 Assays for Cellular Validation

- Coverglass slides (VWR)
- Tecan Infinite M Plex plate reader (Tecan, Switzerland)
- Axio Examiner.Z1 confocal microscope (Carl Zeiss, Cambridge, UK)
- Nikon Eclipse 90i microscope (Nikon, Japan).
- ImageJ Software/FIJI
- Scalpel
- Tweezers

## 3 Methods

### 3.1 Tubular Scaffold Fabrication

Tubular scaffolds were fabricated using a previously reported two step method: melt-extrusion 3D printing of a PCL backbone followed by the integration of a freeze-dried collagen hyaluronic acid bilayer (CHyA-B).^37^

#### 3.1.1 Design and Printing of PCL Backbone

Custom, python generated G-code along with fused deposition modeling 3D printing was used to produce two geometries from 37 kDa PCL; a 360° tubular backbone and a 288° c-shaped backbone which mimics native tracheal cartilage gaps. Each design comprised two concentric rings with 2 mm spacing joined by 5 radial spokes. Each design was printed using a Biobot 1 printer (Allevi, USA) using a 25G stainless-steel nozzle at 100 °C, 90 psi, and 260 mm/min. Each printed scaffold consisted of 40 0.25 mm thick layers of extruded polymer, with an inner diameter of 9.6 mm and an outer diameter of 12 mm.

#### 3.1.2 Preparation of CHyA Films and Slurry

Collagen and hyaluronic acid (CHyA) films were prepared using a suspension of 0.5% w/v type I bovine tendon collagen and 0.044% w/v hyaluronic acid (HyA) in 0.5 M acetic acid (AcOH). CHyA slurry was blended at 4 °C and 15,000 rpm for 3.5 h, after which it was degassed to remove air bubbles introduced during the homogenization process. The CHyA slurry was pipetted onto 6 × 6 cm^2^ polytetrafluoroethylene (PTFE) plate to produce thin transparent CHyA films.

#### 3.1.3 Assembly, Molding and Freeze-Drying

The 3D printed backbones were treated for 4 h with 3 M NaOH solution in Dulbecco’s phosphate-buffered saline (DPBS) to increase wettability and increase collagen infiltration. The scaffolds were combined with CHyA using a custom stainless-steel mold that consisted of 16 pegs of 26 mm in height and 7.8 mm diameter in the bottom plate. The top plate consisted of the respective 16 holes 14 mm wide to allow for the development of tubular scaffolds. CHyA films were cut into 26 × 30 mm strips, rehydrated for 2 h in 0.5 M AcOH and wrapped around each peg of the mold. Films were left overnight under an airflow in a fume hood to allow for dehydration of the films and attachment to the pegs. The following day, 1 mL of CHyA slurry was pipetted into the wells of the metal mold while the 3D printed constructs were gently dropped into the slurry and allowed to sink. The combination was incubated for 2 h at room temperature (RT) to allow for film rehydration and combination with the CHyA slurry, and the scaffolds were thereafter freeze-dried using a customized anneal cycle that initially froze samples to −20 °C before heating to −10 °C and holding for 24 h prior to sublimation.^14^

#### 3.1.4 Sterilization and Cross-Linking

Prior to cellular experiments, scaffolds were sterilized in an ethylene oxide Anprolene AN74 Series gas sterilizer and chemically cross-linked. First, scaffolds were hydrated in 2 mL of DPBS for 15 min and cross-linked for 2 h under UV light using 2 mL of 1-ethyl-3-(3-(dimethylamino)­propyl)­carbodiimide (EDAC) solution which consisted of 6 mM EDAC per gram of scaffold and *N*-hydroxysuccinimide (NHS) in a 5:2 molar ratio of EDAC:NHS.^38,39^ Scaffolds were washed with DPBS to remove any residual toxic product and stored in DPBS at 4 °C prior to use.

### 3.2 Tubular Scaffold Characterization

#### 3.2.1 Mechanical Testing

Tubular and c-shaped PCL-CHyA-B (t/c-PCL-CHyA-B) scaffolds were mechanically characterized with a mechanical testing machine Z050. To evaluate the mechanical strength of the different 3D printed designs, scaffolds were tested by using longitudinal and radial compression with a 50N load cell and 15% strain. The longitudinal compressive modulus was then calculated by using the stress–strain curves. Briefly, the modulus (E) in MPa was calculated as the slope of the stress–strain curve. Compressive Young’s modulus was defined as the applied force (N) per surface area of each sample (m^2^) and strain was calculated as percentage of displacement of the sample during compression. Due to the change in surface area while performing radial compression, the maximum peak load in N was used to measure the construct performance. For radial compression of c-shaped constructs, two positions were employed: the opening facing down parallel to the direction of the force applied (down) or facing to the side (side), perpendicular to the applied force. To further assess the strength of the scaffolds, they were subjected to 15% strain at 20 cycles per minute (0.33 Hz) for a total of 250 cycles in order to mimic the fast-breathing rate observed in humans. Differences between cycles 1 and 250 and between the different designs and polymers were then evaluated. For c-shaped designs, the opening was oriented to the side, perpendicular to the direction of the applied force.

#### 3.2.2 Scaffold Ultrastructure Characterization

To evaluate the porosity of t-PCL-CHyA-B scaffolds, pore size analysis was carried out using a JB-4 Embedding Kit and stained using 0.5% (v/v) toluidine blue solution in distilled water. To estimate the mean pore diameter, scaffolds from different 3D printing and freeze-drying batches were analyzed with a previously described technique.^11,40^ Scaffolds were hydrated overnight in 2 mL of DPBS and fixed using 10% v/v neutral buffered formalin solution. Briefly, scaffolds were incubated with 2 mL of DPBS:formalin 1:1 for 1 h at RT and incubated overnight at 4 °C. Scaffolds were then dehydrated with distilled water for 30 min twice, 50% v/v ethanol for 30 min, 70% v/v ethanol for 1 h 30 min, 95% v/v ethanol for 1 h 30 min, and 100% v/v ethanol for 2 h 15 min. Following rehydration, samples were equilibrated for 12 h at 4 °C using an equilibration solution and were incubated with an infiltration solution for 8 h at 4 °C three times. Infiltration solution was obtained by dissolving benzoyl peroxide in JB-4 Embedding Solution A to a final concentration of 12.5 mg/mL. Equilibration solution was a 1:1 dilution of the infiltration 144 solution and 100% v/v ethanol. After the infiltration steps, the scaffolds were embedded in JB-4 molds using 2 mL of embedding solution which consisted of a 1:25 dilution of JB-4 Embedding Solution B in infiltration solution. Molds with samples were stored at 4 °C until further processing. Sections of 10 μm width from embedded scaffolds were obtained by using a RM2255 automated microtome. Ten sections per scaffold were obtained comprising a total depth of 1000 μm of the scaffold. Sections were then stained using 0.5% v/v toluidine blue solution in distilled H_2_O. Images were obtained using a Nikon Eclipse 90i microscope and a magnification of 10x. Moreover, collagen infiltration within the 3D printed construct was studied by using scanning electron microscopy (SEM) imaging. Imaging of the scaffolds was performed by using a Tescan Mira XMU scanning electron microscope. Images were captured at 5 kV using secondary electron mode, taken at a working distance between 12 and 18 mm.

### 3.3 Manufacture of 3D Printed Accessory Parts for Cellular Seeding

Polylactic acid (PLA) based 3D printed accessory parts were manufactured using an Allevi Biobot 1 3D printer (3D Systems, USA) in order to selectively seed each layer of the scaffold with the cellular population of interest. The goal was to seed the outer layer (OL) of the scaffold using Wi38 fibroblast to mimic the supporting tissues found in the outermost layers of the trachea while the inner layer (IL) was to be populated with Calu-3 cells, representative of the tracheobronchial epithelium.^41^ Two different accessory designs were manufactured, one for Wi38 seeding (Figure S2A,B) and another for Calu-3 seeding (Figure S2C,D). The Wi38 design was developed to expose the OL of the scaffold for cellular seeding while blocking attachment of Wi38 to the IL. It consisted of two parts (top and bottom) with a base of 26.5 mm in diameter and 7 mm in height to fit inside a 50 mL conical tube. The top bottom part had a hollow rod (10 mm in height, outer diameter = 5 mm, inner diameter = 2.4 mm) which allowed positioning and locking with the top part that had a solid rod 2 mm in diameter. Similarly, two parts (top and bottom) formed the Calu-3 accessory parts, with a base similar to that of the Wi38 designs. The bottom design consisted of a hollow rod (10 mm in height, outer diameter = 15 mm, inner diameter = 14 mm) to allow placement of the t-PCL-CHyA-B scaffold, which was then locked in place with a wider rod in the top part (10 mm in height, outer diameter = 17 mm, and inner diameter = 15.2 mm).

### 3.4 Cell Culture and Cell Seeding

#### 3.4.1 Cell Selection and Culture Medium

The Calu-3 bronchial epithelium cell line was cultured in T175 tissue culture flasks following reinvention from liquid nitrogen storage. Calu-3 cells were seeded at a density of 1 × 10^6^ cells/flask and were passaged when they reached confluency using 0.25% Trypsin-EDTA solution. Calu-3 cells were cultured in Dulbecco′s Modified Eagle′s Medium/Nutrient Mixture F-12 Ham medium supplemented with 10% v/v FBS and 100 U/mL penicillin/streptomycin; this was referred to as Calu-3 medium. Cells were used between passages 20–38. The Wi38 human embryonic lung fibroblast cell line (ATCC) was cultured in T175 tissue culture flasks following revival. Wi38 cells were seeded at a density of 1 × 10^6^ cells per flask and were passaged when they reached confluency. Wi38 cells were cultured in Eagle’s minimal essential medium supplemented with 10% v/v FBS, 2 mM l-glutamine, 100 U/mL penicillin/streptomycin, and 1 mM sodium pyruvate; this was referred to as Wi38 medium. Cells were used between passages 18–24. Coculture medium used was a 1:1 mixture of Calu-3:Wi38 medium. Unless otherwise stated, all cell culture and incubation steps were performed at 37 °C and 5% CO_2_ in a humidified atmosphere. Only the t-PCL-CHyA-B scaffolds were used for cellular experiments.

#### 3.4.2 Wi38 Monoculture Seeding and Culture

t-PCL-CHyA-B tubular scaffolds were used to establish the optimal seeding density and conditions for Wi38 cellular seeding in the OL of the scaffold. Following chemical cross-linking, scaffolds were prewarmed with 2 mL of Wi38-medium. Conical tubes were prepared for cellular seeding by placing the Wi38 bottom accessory part and the t-PCL-CHyA-B scaffold into the tube (Figure 1A). Cell suspension containing the desired concentration of Wi38 cells was pipetted into the conical tube while the scaffold was held in place using tweezers. The top accessory part was then placed to lock the inner layer (IL) of the scaffold from cellular seeding, the conical tube was closed and placed in the roller for cellular seeding under rotation (1 rpm) for 2 h at 37 °C and 5% CO_2_. Three different cell seeding densities were used, as shown in Table 1, which were obtained from previous expertise from our group using CHyA-B scaffolds as a model of the tracheobronchial regions (12, 40). Cells were grown for 7 days and fed every 2 days with 10 mL of fresh Wi38 medium prior to assessing fibroblast growth on the scaffolds.

**Figure 1:**
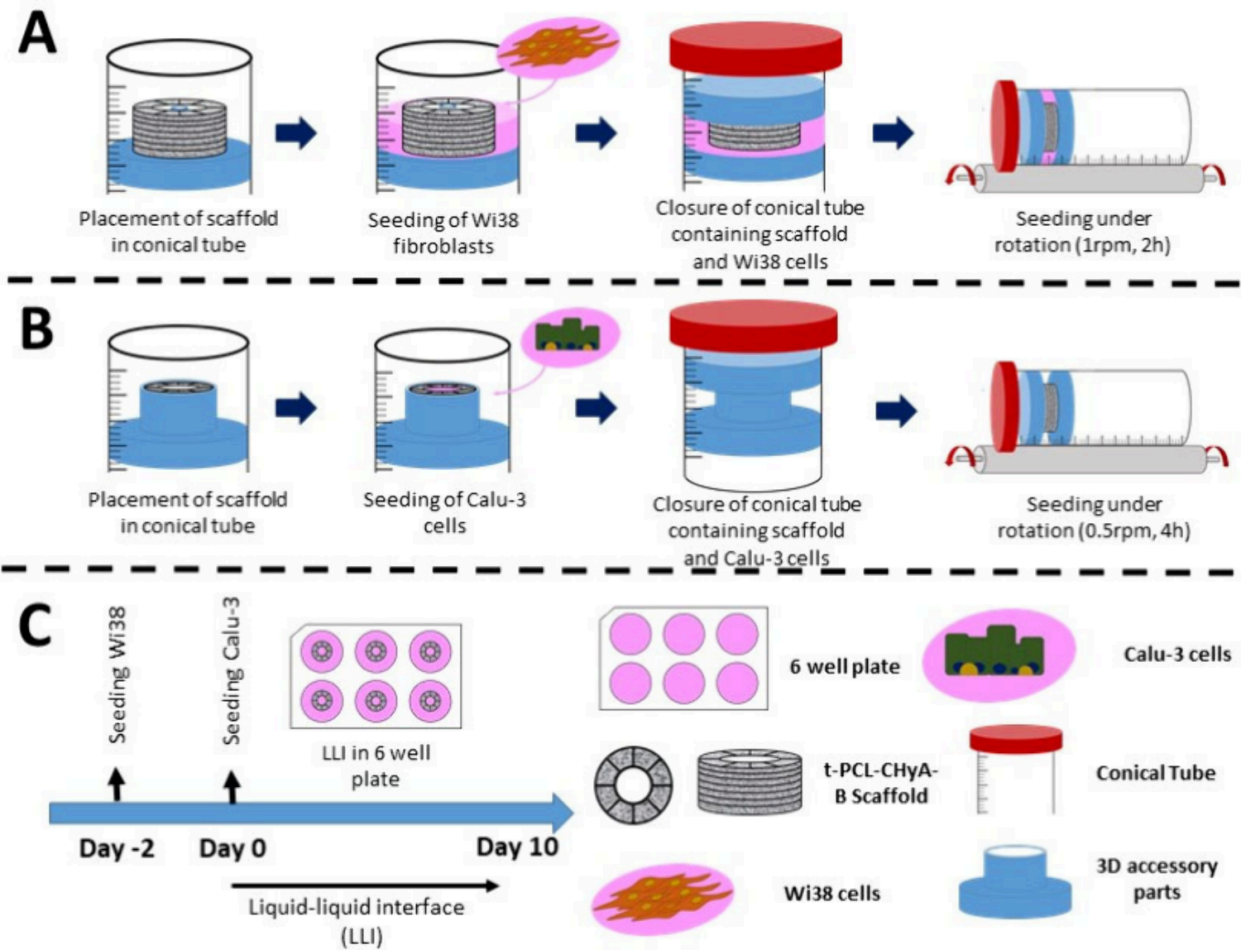
Seeding approaches for tubular bilayered PCL-Collagen and Hyaluronic (t-PCL-CHyA-B) scaffolds: (A) Wi38 monoculture, (B) Calu-3 monoculture, and (C) coculture.

**Table 1:** Seeding Densities Used to Populate the Outer Layer (OL) of Tubular Bilayered PCL-Collagen and Hyaluronic (t-PCL-CHyA-B) Scaffolds Using Wi38 Lung Derived Fibroblasts

**Seeding Density**	**Area (cells/cm** ^**2**^ **)**	**Scaffold (cells/scaffold)**
Density A	150.000 = 1.5 × 10^5^	0.625 × 10^6^
Density B	300.000 = 3 × 10^5^	1.25 × 10^6^
Density C	600.000 = 6 × 10^5^	2.5 × 10^6^

#### 3.4.3 Calu-3 Monoculture Seeding and Culture

t-PCL-CHyA-B tubular scaffolds were also used to optimize the epithelial seeding process (cell density and conditions) using Calu-3 cells in the inner layer (IL) of the scaffolds. Following chemical cross-linking, scaffolds were prewarmed with 2 mL of Calu-3 medium. Conical tubes were prepared for cellular seeding by placing the Calu-3 bottom accessory part and the t-PCL-CHyA-B scaffold in the tube (Figure 1B). Cell suspension containing the desired concentration of Calu-3 cells was pipetted into the IL of the scaffold, and the top accessory part was then placed to lock the OL of the scaffold from cell seeding. The conical tube was closed and placed in the roller for cellular seeding under rotation (0.5 rpm) for 4 h at 37 °C and 5% CO_2_. Three different cell densities were used as shown in Table 2, optimized from previously reported Calu-3 culture studies conducted on scaffolds for tracheal repair.^13,42,43^ To investigate the effect of the seeding accessory parts, some scaffolds were seeded using 15 mL conical tubes without the accessories. Scaffolds were placed in conical tubes that had been filled with melted PCL until the 12 mL mark for tight placement of scaffolds during the seeding process. Cells were grown for 10 days and fed every 2 days with 10 mL of fresh Calu-3 medium prior to assessing epithelial growth on the scaffolds.

**Table 2:** Seeding Densities Used to Populate the Inner Layer (IL) of Tubular Bilayered PCL-Collagen and Hyaluronic (t-PCL-CHyA-B) Scaffolds Using Calu-3 cells

**Seeding Density**	**Area (cells/cm** ^**2**^ **)**	**Scaffold (cells/scaffold)**
Density A	1.25 × 10^5^	0.3925 × 10^6^
Density B	2.5 × 10^5^	0.785 × 10^6^
Density C	5 × 10^5^	1.57 × 10^6^

#### 3.4.4 Coculture and Liquid–Liquid Interface (LLI) Conditions

To determine the ability of the seeding approach to support coculture seeding, Wi38 cells were seeded on the PCL-CHyA-B scaffolds, as described in Section 3.4.2 on day 2 of culture using Density A (6 × 10^5^ cells/cm^2^) (Figure 1C). Scaffolds were then incubated for 48 h using 10 mL of Wi38 medium and seeded with Calu-3 cells on day 0 using Density A (1.25 × 10^5^ cells/cm^2^) for either 4 or 2 h under rotation. Scaffolds were then kept under liquid–liquid interface (LLI) conditions for 10 days in 10 mL of coculture medium, exchanging medium every second day. On day 10 of culture, scaffolds were processed and the cell growth on the scaffolds was evaluated as outlined in Section 3.5.

**Figure 2:**
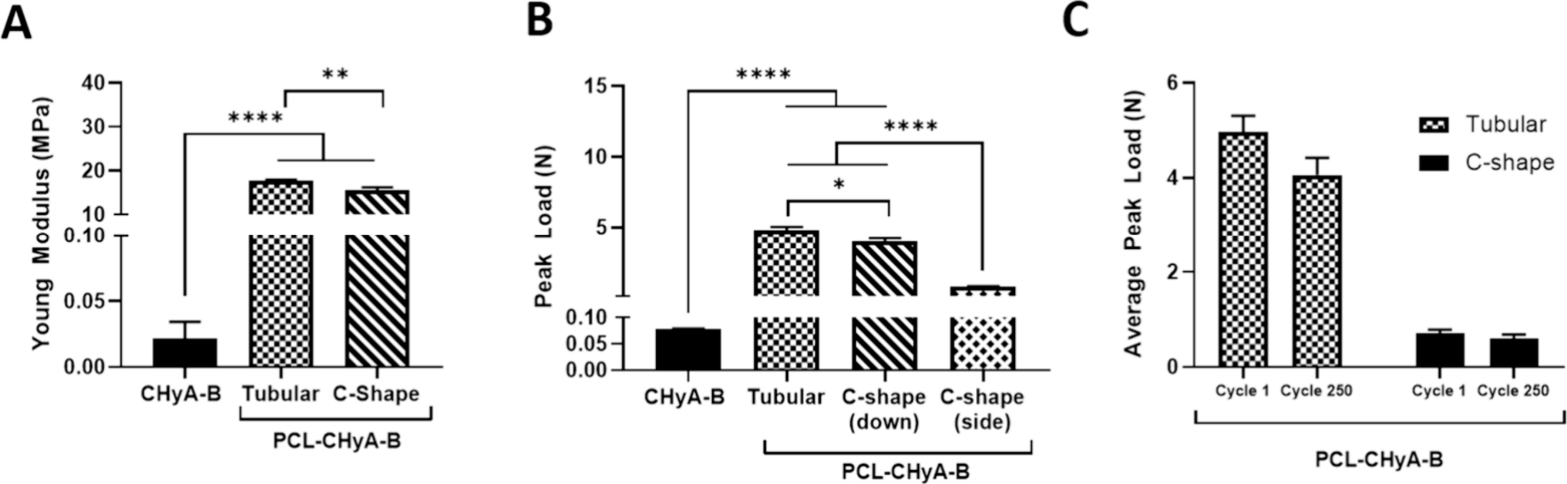
Mechanical characterization of tubular and c-shaped bilayered PCL-Collagen and Hyaluronic (PCL-CHyA-B) scaffolds. (A) Longitudinal compression. (B) Radial compression performed using a 50 N and 20% strain. (C) Cyclic loading performed using a 50 N load cell and 20% strain. Results displayed as mean ± SEM (*n* = 3, * *p* < 0.05, ** *p* < 0.01, **** *p* < 0.0001).

### 3.5 Assays for Cell Seeding Characterization

#### 3.5.1 Metabolic Activity

Cell metabolic activity on t-PCL-CHyA-B scaffolds was quantified using AlamarBlue Cell Viability Reagent as per manufacturer instructions. Briefly, for Wi38 monoculture experiments, scaffolds were transferred on days 1 and 5 of culture to a 24 well plate containing 1.5 mL of 1X alamarBlue diluted in culture media and incubated for 2 h prior to measuring fluorescence using 545 nm excitation and 590 nm emission wavelengths in a Tecan Infinite M Plex plate reader. At day 7 of culture, each scaffold was disassembled by separating the OL from the IL layers and both sections were incubated separately with 1.5 mL of 1X alamarBlue, respectively. Similarly, for Calu-3 monoculture and coculture experiments, metabolic activity from the whole scaffold was measured at days 2 and 5 of culture, while at day 10 each layer (OL and IL) was independently assessed to evaluate cellular growth on the different layers of the scaffold.

#### 3.5.2 DNA Quantification

Scaffolds were processed at day 7 (Wi38 monoculture) or day 10 (Calu-3 monoculture and coculture) to quantify double-stranded DNA (dsDNA) from the scaffolds using a Quant-iT PicoGreen dsDNA Kit. Briefly, the IL and OL of the scaffold were separated carefully using tweezers, rinsed in DPBS and submerged in 1 mL of lysis buffer, which consisted of 1% v/v Triton X-100 and 0.2 M Na_2_CO_3_ in distilled H_2_O and freeze thaw three times at −80 °C.

#### 3.5.3 Cell Growth on the OL of the Scaffold

Cell growth on the OL of the scaffold was evaluated on day 7 (Wi38 monoculture) or day 10 (Calu-3 monoculture and coculture) to assess cell proliferation. Films were detached from the porous layer of the scaffold (OL) and rinsed with DPBS for 5 min. Small sections from the porous layer were cut using a scalpel and tweezers to hold the scaffold in place and fixed using 1.5 mL of 10% v/v neutral buffered formalin solution at RT for 10 min. Following every step, scaffolds were washed in DPBS for 3 min at RT. Scaffolds were then incubated with 0.1% v/v Triton X-100 in DPBS for 5 min to permeabilize cells, followed by 1% w/v bovine serum albumin (BSA) in DPBS for 30 min. Cells growing in the OL of the scaffold were stained using 1:600 phalloidin (Phalloidin–Tetramethylrhodamine B isothiocyanate) diluted in 1% v/v BSA in DPBS for 20 min followed by a 10 min incubation with 1:1000 DAPI readymade solution diluted in 1% BSA in DPBS. Scaffolds were then stored at 4 °C until imaging using an Axio Examiner.Z1 confocal microscope (Carl Zeiss, Cambridge, UK). Images were processed and examined using FIJI.^44^

#### 3.5.4 Epithelial Coverage of the inner layer (IL) of the Scaffold

At day 7 (Wi38 monoculture) or day 10 (Calu-3 monoculture and coculture), detached films (IL) were processed to quantify the cell coverage of the film. Scaffolds were rinsed in DPBS and fixed using 1.5 mL of 10% v/v neutral buffered formalin solution at RT for 10 min. Following every step, films were washed in DPBS for 3 min under a rotation at 5 rpm. All of the following steps were performed under rotation at RT. Films were then incubated with 0.1% v/v Triton X-100 in DPBS for 5 min, followed by 1% w/v bovine serum albumin (BSA) in DPBS for 30 min. Films were stained using phalloidin and DAPI as per Section 3.5.3. Films were then placed on cover glass slides, and a drop of Invitrogen ProLong Glass Antifade Mountant was used to cover the films and sealed with clear nail polish. Epithelial coverage on the films was evaluated by imaging the films with a Nikon Eclipse 90i microscope and estimating the cell coverage using FIJI-based code developed by Brenton Cavanagh (Cellular and Molecular Imaging Core, RCSI) (Supplementary Info). Images were obtained using 4× and 10× magnification, although whole film images using 4× magnification were used for estimating the epithelial coverage. Briefly, the code calculated the area in cm^2^ of the film by using the DAPI stain which was also able to stain the CHyA film while the area in cm^2^ covered by cells was estimated using the phalloidin stain. Finally, the coverage of the film was obtained using the following equation:

$$\mathrm{Cellular Coverage}\;\left(\%\right)=\frac{\mathrm{Area stained with Phalloidin}\;\left({\mathrm{cm}}^{2}\right)}{\mathrm{Area stained with DAPI}\left({\mathrm{cm}}^{2}\right)}\times 100$$

## 4 Validation and Expected Results

### 4.1 Scaffold Manufacture and Characterization

Previous work in this group^37^ resulted in the development of a scaffold consisting of a 3DP backbone combined with collagen and hyaluronic acid (CHyA-B), which were designed to address the dual challenges of mechanical robustness and vascularization in tracheal tissue engineering. It was shown that the inclusion of a 3DP PCL framework significantly enhanced the mechanical properties of the CHyA scaffolds. Scaffolds were successfully manufactured into a tubular and c-shaped structure suitable for tracheal regeneration by combining extrusion-based 3D printing of PCL to prepare a polymeric backbone and a previously described freeze-drying methodology to manufacture CHyA scaffolds.^13,42^ 3D printed tubular constructs consisted of 40 layers with a 4.8 and 6.6 mm inner and outer radius interconnected with 5 spokes in each layer and combined with the CHyA slurry and CHyA films resulting in a multilayered scaffold with an inner film (IL) and a highly porous outer layer (OL) (Figure S1).

One key aspect hampering the development and translation of tracheal substitutes is the lack of standard mechanical testing protocols and the scarcity of mechanical data from human samples (5). The complex nature of the trachea means it is difficult to establish the mechanical requirements needed, leading to testing inconsistencies and difficulty in comparing results between different studies.^45^ Tracheal samples are difficult to obtain, therefore partial sections of the organ are tested, failing to recapitulate whole organ mechanics.^46^ Here, we performed compression in both longitudinal and radial directions (Figure 2A,B). The inclusion of PCL proved to significantly increase the strength of the scaffold in comparison to nonreinforced CHyA-B scaffolds for both the c-shaped and the tubular design. Longitudinal compression did not show significant differences between the tubular and c-shaped designs (Figure 2A). The reported compressive Young’s modulus for trachea constructs, in the range of 1–2 MPa in porcine and rabbit models, is lower than that reported here.^47,48^ However, it is important to consider the difference between human and animal samples, the testing setup used, and whole-organ versus section testing. 3D printing also allows for rapid design modifications that can alter the mechanical properties to match the desired requirements. Similar to the improvements observed in longitudinal compression tests, radial testing demonstrated that PCL-CHyA-B scaffolds significantly increased the supported peak load compared to nonreinforced ones (Figure 2B). The average peak load (5 N) reported was lower than the compression strength observed in porcine, canine and caprine tracheal samples (8–15 N), but closer to the one reported in human tracheal samples (0.55–2.32 N).^49^ Fatigue testing was employed to evaluate whether the designs were able to withstand constant mechanical loading to mimic the respiration force patterns being continually exerted on tracheal tissues. Both designs were able to withstand the cyclic loading of 250 cycles, showing no significant decrease when compared to the respective cycle 1 and demonstrating the stable mechanical properties overtime of the PCL-CHyA-B scaffolds (Figure 2C). From here on, tubular designs were used only for further experiments. Comparison to pre-existing data on radial compression for human trachea and the ability of 3D printed constructs to withstand repeated cycles of applied force demonstrate that 3D printed polymer reinforced scaffolds could potentially offer a TET approach with the desired mechanical strength.

The formation of a porous network of collagen and hyaluronic acid within the 3D printed construct would allow for the seeding of the outer portion of the tubular scaffolds with relevant cells, e.g., cartilage formation as previously demonstrated with other scaffolds aiming at tracheal repair.^50,51^ Pore size analysis using toluidine blue and SEM imaging was carried out to ensure that the CHyA slurry was effectively incorporated into the backbone of the tubular PCL-CHyA-B scaffolds and to determine if pore size was affected. A similar pore morphology and distribution was observed in the CHyA-B tubular scaffolds compared to nonreinforced scaffolds, also confirming successful infiltration of the CHyA slurry within the 3D printed constructs (Figure 3A–D). Pore size analysis revealed a significant increase to above 150 nm in pore diameter for reinforced tubular scaffolds compared to nonreinforced ones (Figure 3E). The presence of the 3D printed tubular scaffolds increased the mean pore diameter of the collagen sublayer from 114.3 μm in CHyA-B tubular scaffolds to 161.8 μm in PCL-CHyA scaffolds. The obtained pore sizes are in the range of 100–200 μm, which has been shown to promote proliferation and differentiation of cartilage tissues which would ultimately be expected to grow on the porous outer sublayer of this tubular scaffolds.^52,53^

**Figure 3:**
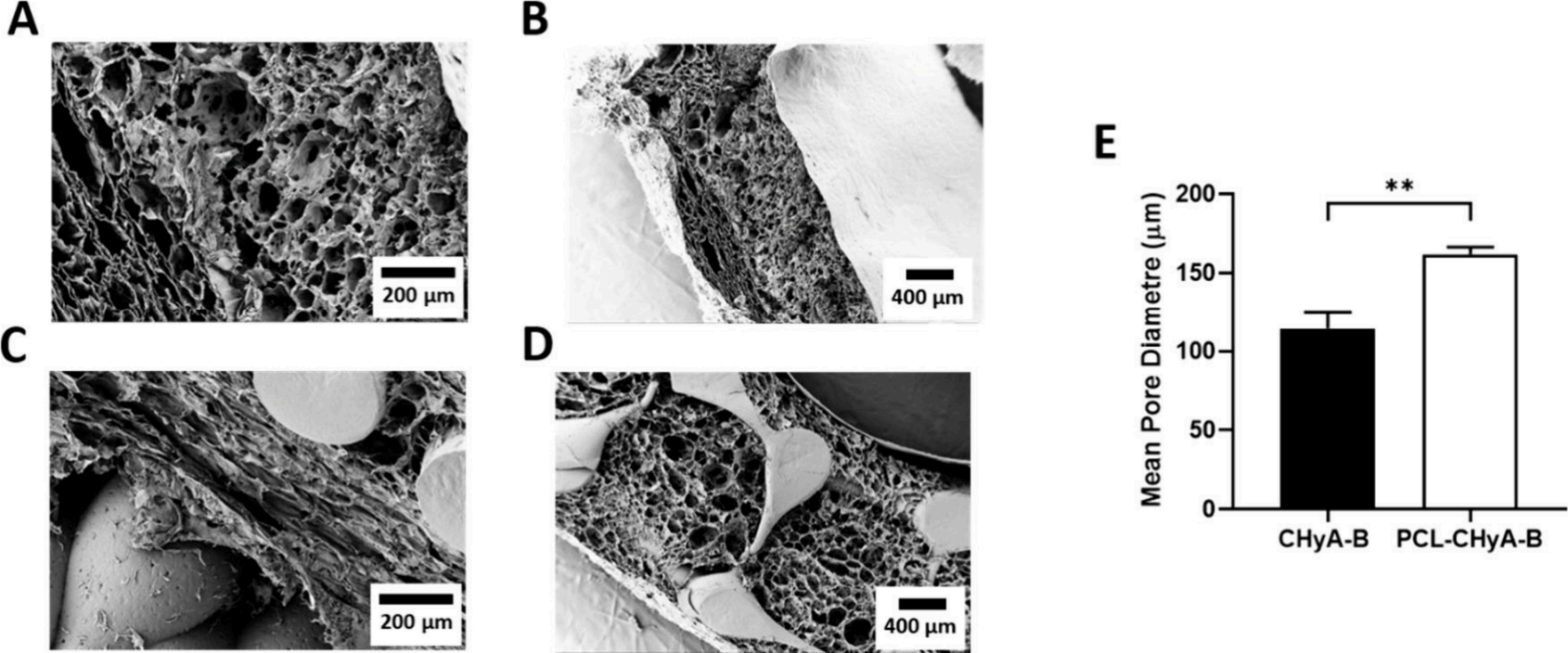
Ultrastructure of tubular PCL hyaluronic acid t-PCL-CHyA-B scaffolds. Scanning electron microscopy of t-CHyA-B (A,B) and t-PCL-CHyA-B (C,D) scaffolds. Mean pore diameter of t-CHyA-B and t-PCL-CHyA-B scaffolds estimated using Toluidine Blue Assay (E). Results displayed as mean ± SEM (*n* = 3, **p < 0.01).

### 4.2 Cellular Growth in the Inner Layer (IL) of the Scaffold (Calu-3 Monoculture)

To demonstrate the ability of the t-PCL-CHyA-B scaffolds to support the growth of respiratory epithelium on the IL, Calu-3 cells, a respiratory epithelial cell line, were seeded onto the scaffold using a custom-designed 3DP accessory that selectively exposed the IL for attachment while shielding the OL. In this setup, the scaffold was placed within the accessory, which securely held it in place and allowed the seeding solution to be applied directly to the IL while preventing overflow onto the OL. A negative control group involved seeding the scaffolds without the accessory, allowing for unrestricted cellular attachment to all of the scaffold layers. As shown in Figure 4A, the use of the 3DP accessory enabled the successful attachment and growth of Calu-3 cells in the IL of the scaffold, with minimal growth observed in the OL compared to the negative control where no accessory was used, demonstrating the efficacy of the accessory in achieving spatially selective seeding.

**Figure 4:**
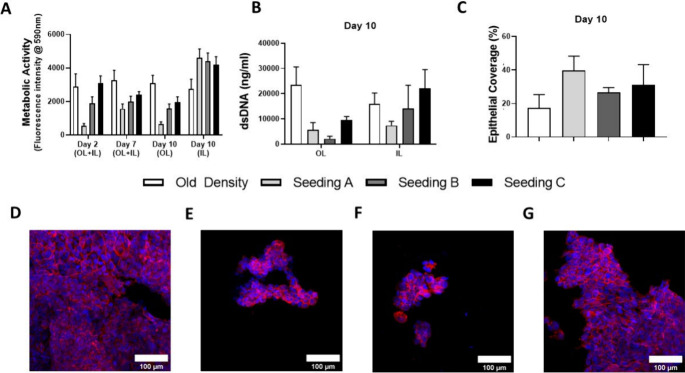
Seeding of the IL of tubular bilayered PCL-Collagen and Hyaluronic acid (t-PCL-CHyA-B) scaffolds using Calu-3 cells at several densities; seeding A (1.25x10^5^ cells/cm^2^), seeding B (2.5x10^5^ cells/cm^2^) and seeding C (5x10^5^ cells/cm^2^). (A) Cell metabolic activity of Calu-3 cells on the OL and IL on days 2, 7, and 10. (B) DNA quantification on the OL and IL at day 10. (C) Coverage of the IL by Calu-3 cells at day 10. Representative images of Calu-3 cells growing on the OL without using 3D printed accessories (D) and using three seeding densities together with 3D printed accessories: (E) 1.5 × 10^5^ cells/cm^2^, (F) 2.5 × 10^5^ cells/cm^2^ and (G) 5 × 10^5^ cells/cm^2^. Imaging was performed using a Axio Examiner.Z1 confocal microscope on cells stained with DAPI (blue) and phalloidin (red) to show the nuclei and actin filaments, respectively. Results displayed as mean ± SEM (*n* = 3, * *p* < 0.05, ** *p* < 0.01, *** *p* < 0.001).

Furthermore, the smallest seeding density (1.5 × 10^5^ cells/cm^2^) yielded optimal cellular attachment and growth on the IL while significantly reducing cellular attachment in the OL compared to other seeding densities. This was confirmed by measuring the DNA content of the scaffolds after 10 days in culture (Figure 4B), which showed a significant increase in the cellular coverage of the IL compared to the negative control which was seeded without using accessories (Figure 4C). Confocal microscopy images of DAPI and phalloidin stained cells further validated these findings, showing a limited cellular presence in the OL when scaffolds were seeded with 1.5 × 10^5^ cells/cm^2^ using the 3DP accessories (Figure 4E). In contrast, prominent cellular attachment in the OL was observed when the scaffolds were seeded without accessories (Figure 4D). This spatial selectivity was further evident at the higher seeding densities of 2.5 × 10^5^ cells/cm^2^ and 5 × 10^5^ cells/cm^2^ when using the accessories (Figure 4F,G), highlighting the accessory’s role in directing cell attachment primarily to the IL.

The highest coverage of the IL was also observed when a seeding density of 1.5 × 10^5^ cells/cm^2^ was used, with a uniform layer of cells distributed across the surface (Figure 5).

**Figure 5:**
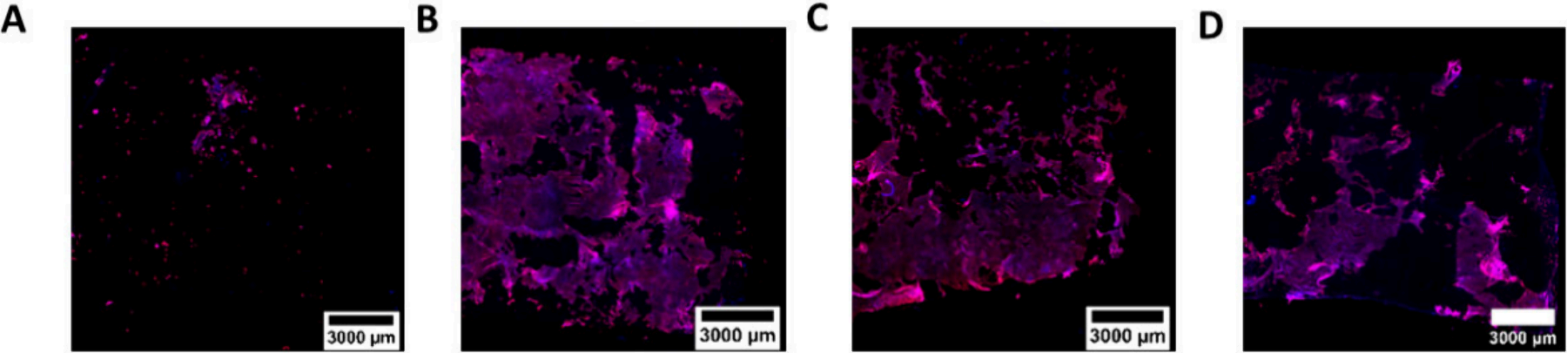
Representative confocal microscopy images of Calu-3 cells growing on the IL of tubular bilayered PCL Collagen and Hyaluronic acid (t-PCL-CHyA-B) scaffolds without using 3D printed accessories (A) and using three seeding densities together with 3D printed accessories: (B) 1.5 × 10^5^ cells/cm^2^, (C) 3 × 10^5^ cells/cm^2^ and (D) 6 × 10^5^ cells/cm^2^. Imaging was performed using an Axio Examiner.Z1 confocal microscope on DAPI-stained nuclei (blue) and phalloidin-stained actin filaments (red).

These results demonstrate the feasibility of the 3DP accessory in achieving targeted seeding of respiratory epithelial cells on the IL of the scaffold, and this approach was subsequently used for further coculture experiments.

### 4.3 Cellular Growth in the OL of the Scaffold (Wi38 Monoculture)

The seeding method was validated to demonstrate its ability to achieve spatially controlled distribution of relevant cell types on tubular scaffolds, using Wi-38 lung-derived fibroblasts as an example for subepithelial tracheal tissues. This system aimed to maximize selective attachment and growth of fibroblasts in the outer layer (OL) of the scaffold while minimizing unintended seeding on the inner layer (IL), which is designed for the growth of respiratory epithelial cells.

Wi-38 cells were seeded onto t-PCL-CHyA-B scaffolds at three densities (1.5 × 10^5^, 3 × 10^5^ and 6 × 10^5^ cells/cm^2^) under rotation with a 3DP seeding chamber designed for targeted seeding of the outer layer of the scaffolds—where surrounding tracheal tissues will interact with the scaffold—while aiming to minimize attachment of cells to the inner layer (film)—designed to support the growth of a respiratory epithelium. The chamber ensured efficient cell seeding of the OL while preventing significant cell attachment to the IL. Cell metabolic activity, DNA quantification, and cell coverage were assessed and confirmed successful fibroblast attachment and growth predominantly in the OL (Figure 6A,B), with minimal cell presence in the IL (Figure 6C). DAPI and Phalloidin staining of the cell-seeded scaffolds further demonstrated Wi-38 attachment primarily to the collagen-based OL of the scaffold (Figure 6D–F). Seeding density of Wi38 cells at 6 × 10^5^ cells/cm^2^ was shown to result in the highest cell growth, with a significantly increased metabolic activity and DNA content in comparison to the other cell densities used at day 7 of culture (Figure 6A,B). This was confirmed by cell imaging using confocal microscopy (Axio Examiner.Z1 confocal microscope, Carl Zeiss) that showed a typical fibroblast-growth pattern (Figure 6F). To confirm fibroblast coverage primarily in the OL of the scaffold, the collagen-based film of the IL was imaged using DAPI-Phalloidin showing no growth of fibroblasts in the IL (Figure S3A–C). For further coculture experiments, t-PCL-CHyA-B scaffolds were seeded using a concentration of Wi38 fibroblast of 6 × 10^5^ cells/cm^2^.

**Figure 6:**
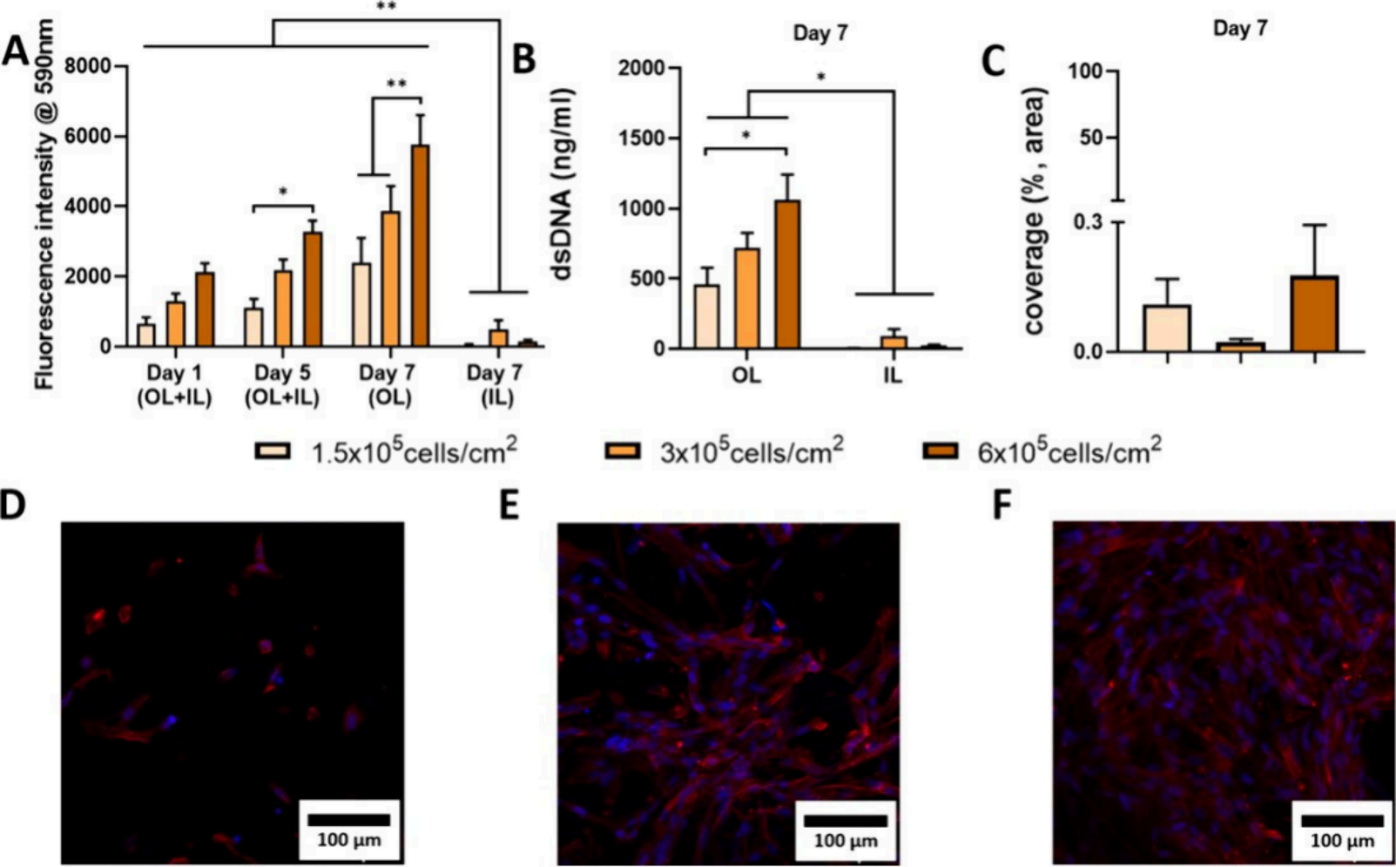
Seeding of the OL of tubular bilayered PCL-Collagen and Hyaluronic (t-PCL-CHyA-B) scaffolds using Wi38 cells. (A) Cell metabolic activity of Wi38 cells on the OL and IL on days 1, 5, and 7. (B) DNA quantification on the OL and IL at day 7. (C) Coverage of the IL by Wi38 cells at day 7. Representative images of Wi38 cells growing on the OL using three seeding densities: (D) 1.5 × 10^5^ cells/cm^2^, (E) 3 × 10^5^ cells/cm^2^ and (F) 6 × 10^5^ cells/cm^2^. Cells were treated with DAPI (blue) and phalloidin (red) to stain the nuclei and actin filaments, respectively. Images were captured via confocal microscopy (Axio Examiner.Z1 confocal microscope). Results displayed as mean ± SEM (*n* = 3, * *p* < 0.05, ** *p* < 0.01).

### 4.4 Establishing a Coculture in t-PCL-CHyA-B Scaffolds Using Seeding Accessories

Tracheal regeneration strategies focused on the use of preseeded implants indicated a critical role for coculture of multiple, relevant cell types on the TET scaffolds to support the development and ultimate clinical use of TET approaches. In the first instance, in vitro cell seeding of scaffolds validates their ability to support the growth of all of the relevant tissue types. However, achieving spatially selective growth of multiple cell types on specific scaffold layers is highly challenging without the use of dedicated tools such as 3DP accessories, which ensure precise exposure of individual layers for targeted cell attachment. In addition, the ability to selectively grow multiple relevant cell types on a multilayered scaffold offers the potential to produce preseeded scaffolds for implantation to enhance tracheal regeneration in the clinical setting.

Here, a method to seed and establish a coculture of Wi38 lung derived fibroblast and Calu-3 bronchoepithelial cells was developed using the seeding densities previously optimized and the 3D printed accessories developed specifically for tubular scaffold seeding. To achieve this, both sets of accessories were used sequentially, with the accessory for the OL employed first to seed the Wi38 cells on the outer layer (at a density of 6 × 10^5^ cells/cm^2^ for 4 h) followed by the accessory for the IL to seed Calu-3 cells on the inner layer, ensuring spatially selective coculture on the scaffold.

After 10 days in culture, the coculture scaffolds showed a significant decrease in overall cell metabolic activity measured in the OL of the scaffold when compared to the Wi38 monoculture (Figure 7A) whereas a significant increase was observed in the IL under coculture conditions (Figure 7A,B). It was hypothesized that the Wi38 cells seeded in the OL were unable to withstand the hypoxic conditions and reduced culture medium flow, while the OL was protected from Calu-3 seeding by using the 3DP accessories. Imaging confirmed this hypothesis, showing extensive growth of Wi38 in the OL under monoculture conditions compared to coculture (Figure S4A,C). Imaging was performed using an Axio Examiner.Z1 confocal microscope with DAPI staining (blue) to label nuclei and phalloidin staining (red) to label actin filaments.

**Figure 7:**
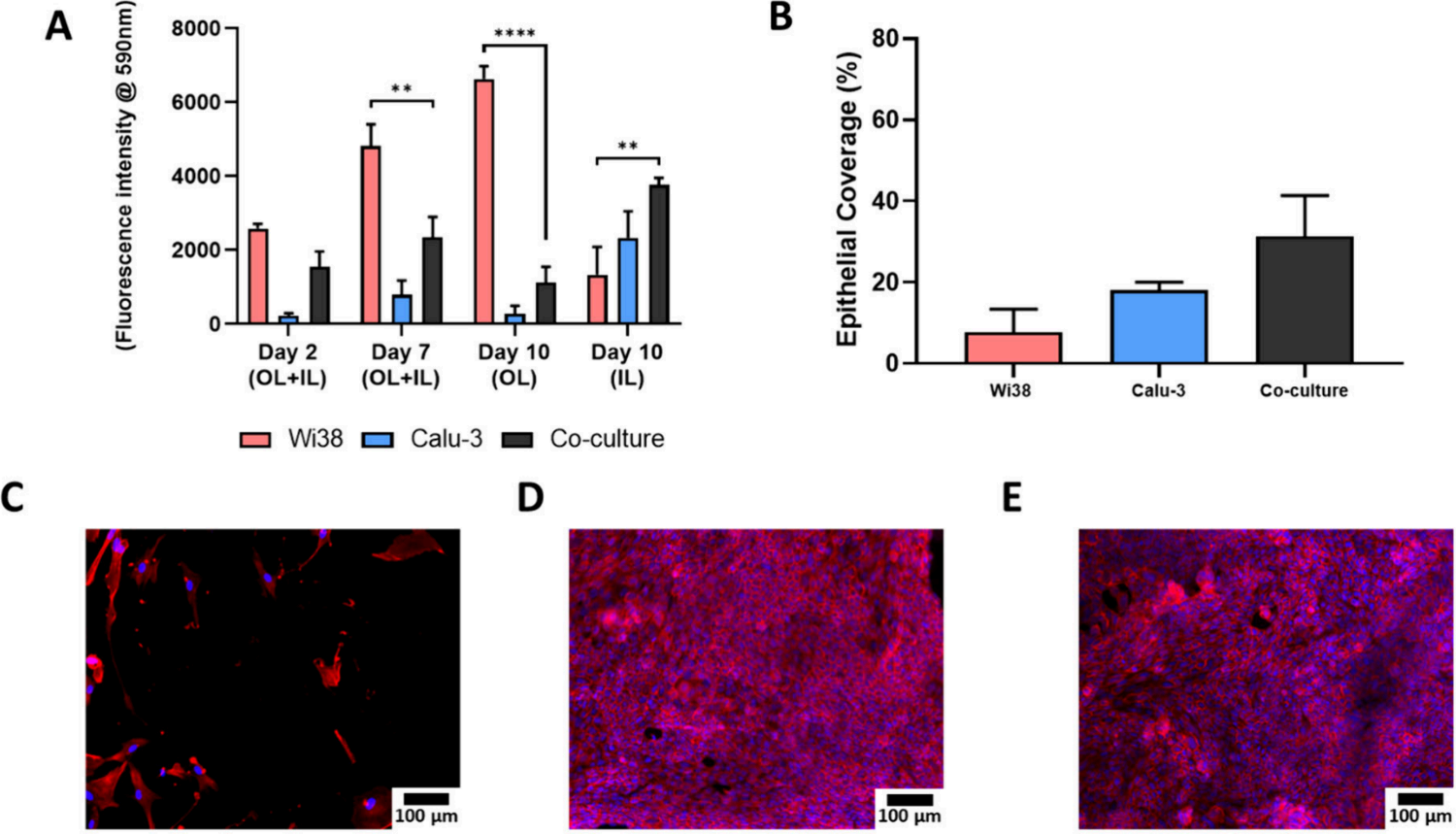
Coculture on tubular bilayered PCL-Collagen and Hyaluronic acid (t-PCL-CHyA-B) scaffolds seeded on the outer layer (OL) with Wi38 cells and the inner layer (IL) with Calu-3 cells for 4h under rotation using 3D printed accessories. (A) Cell metabolic activity at days 2, 7, and 10 of culture. (B) Coverage of the IL at day 10 of culture. Representative images of the IL of t-PCL-CHyA-B scaffolds seeded with (C) Wi38 monoculture, (D) Calu-3 monoculture and (E) coculture using 3D printed accessories. Images were obtained using a Axio Examiner. Z1 confocal microscope. Cells were stained with DAPI (blue) to label nuclei and phalloidin (red) to stain actin filaments. Results displayed as mean ± SEM (*n* = 3, ** *p* < 0.01, **** *p* < 0.0001).

Interestingly, epithelial coverage of the IL appeared higher under coculture conditions compared to monoculture at the same Calu-3 seeding density, as shown in Figure 7B, although the increase was not statistically significant. This suggests that potential crosstalk between the two cell types may support epithelial proliferation. Signs of enhanced differentiation of the epithelial layer under coculture conditions were observed, with cells adopting a more uniform and cohesive layer compared to monoculture, which may be indicative of improved functionality. The growth of Calu-3 cells in the IL under coculture conditions showed no significant difference compared to monoculture as show in Figure 7D,E.

In order to determine the impact of Calu-3 seeding time on the viability of Wi38 cells, the time for Calu-3 cell seeding under rotation was reduced to 2 h. Using this reduced seeding time, similar cell metabolic activity in the OL was observed at day 10 in Wi38 monoculture (Figure 8A). Additionally, the overall cell metabolic activity in the IL was comparable when comparing coculture and Calu-3 culture (Figure 8A). There was no statistical difference between Wi38 monoculture and coculture in the OL by day 10 under these conditions.

**Figure 8:**
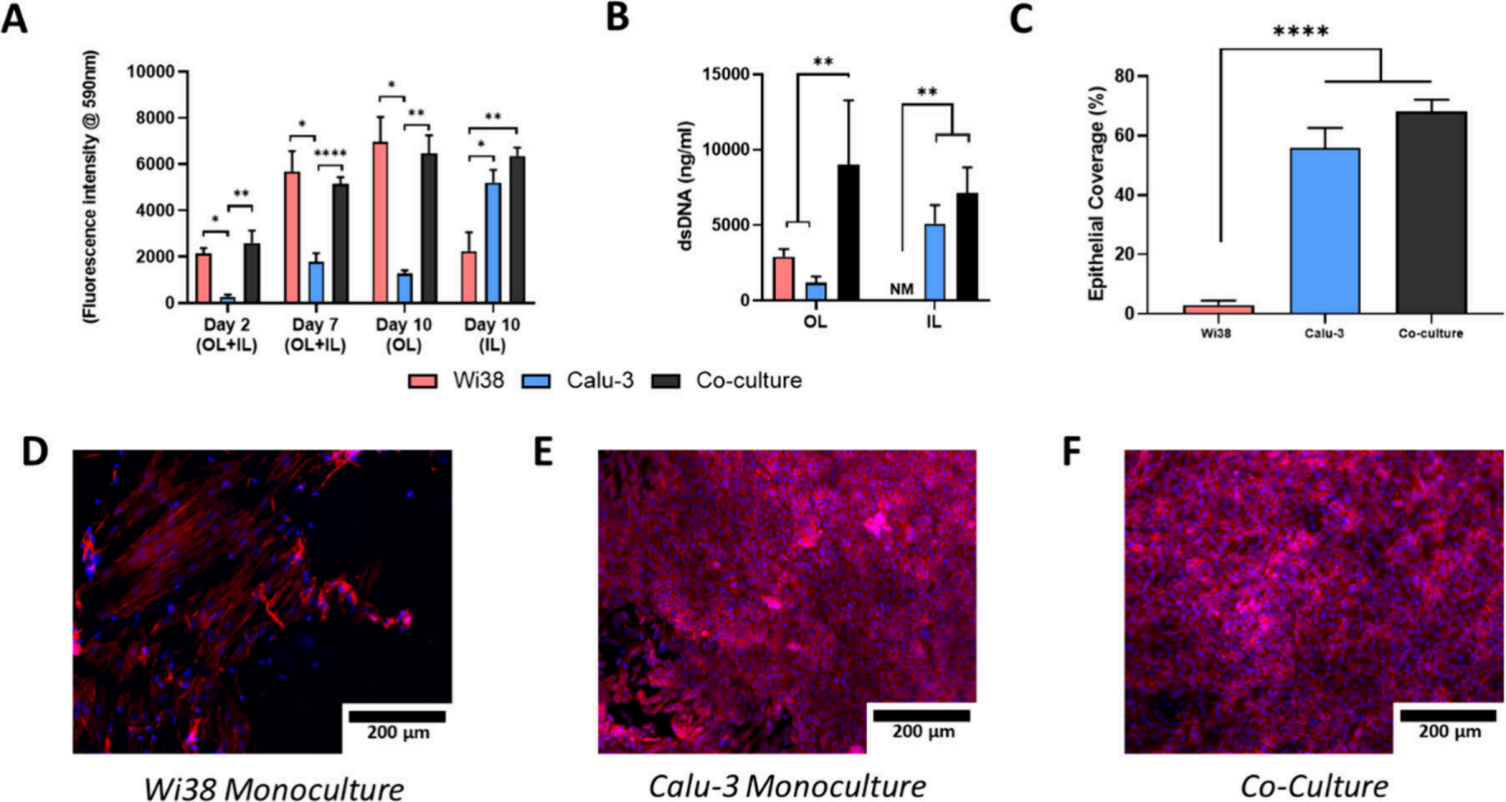
Coculture on tubular bilayered PCL-Collagen and Hyaluronic (t-PCL-CHyA-B) scaffolds seeding the OL with Wi38 cells and the IL with Calu-3 cells for 2 h under rotation. (A) Cell metabolic activity at days 2, 7, and 10 of culture. (B) Coverage of the IL at day 10 of culture. Representative images of the IL of t-PCL-CHyA-B scaffolds: (D) Wi38 monoculture, (E) Calu-3 monoculture, and (F) coculture. Images were obtained using an Axio Examiner Z1 confocal microscope. Cells were stained with DAPI (blue) to label nuclei and phalloidin (red) to stain actin filaments. Results displayed as mean ± SEM (*n* = 3).

This finding was further confirmed using DNA quantification (Figure 8B), which showed a significant increase in epithelial coverage, reaching close to 70% under coculture conditions compared to approximately 30% in previous experiments. However, there was no statistical difference in epithelial coverage between Calu-3 monoculture and coculture conditions.

Moreover, adequate epithelial growth was seen in the IL of the coculture (Figure 8F) after 10 days, along with sustained fibroblast growth in the OL without compromising the efficiency of Calu-3 seeding. This provides evidence for the development of a successful *in vitro* coculture of respiratory cells on t-PCL-CHyA-B scaffolds.

## 5 Conclusion

This study validated a method for fabricating tubular scaffolds with mechanical properties suitable for tracheal regeneration and demonstrated their ability to support spatially selective cell seeding of relevant cell types using a custom-made 3D printed accessory system. We were able to selectively seed respiratory epithelial cells (Calu-3) in the inner layer and lung derived fibroblasts (Wi38) on the outer layer of the scaffolds. This represents a critical step in addressing the challenges of cell seeding on multilayered tubular scaffolds, offering a scalable and customizable approach for tracheal tissue engineering. The ability to establish cocultures on these scaffolds further validates their potential for supporting complex cellular environments in preimplantation strategies, which is a necessary feature for effective tracheal prototypes.^54^

The development of selective seeding protocols for tubular constructs addresses a significant unmet need in tissue engineering. Standard methods such as soak-loading often result in poor spatial control and low cell viability, particularly in multilayered scaffolds.^55,56^ The approach presented here is not confined to specific scaffold compositions such as PCL and collagen but could be adapted to other materials and geometries, broadening its applicability. Beyond tracheal regeneration, this method holds promise for applications in other tubular constructs, such as synthetic blood vessels, urethral scaffolds, and gastrointestinal tissue engineering. These systems often require the targeted growth of specific cell types in distinct layers to mimic the structures and functions of native tissues. The novel cell seeding process outlined here can be scaled up for use on larger tubular constructs by 3D printing larger accessories to match the dimensions of the scaffold, which is a relatively straightforward process using PLA.

Future work should focus on validating this method with primary cells commonly used in tracheal tissue engineering to ensure compatibility with clinically relevant cell types.^57,58^ Incorporating an air–liquid interface culture could further enhance the differentiation of the respiratory epithelium, a critical feature for reducing infection risks in tracheal implants.^43,59,60^ By advancing these methodologies, this study provides a foundation for the clinical translation of tissue-engineered scaffolds requiring preseeding strategies, paving the way for improved patient outcomes in tracheal regeneration and other tubular tissue engineering applications.

## Supplementary Material



## Notes

Additional manufacturing details with photographs of each stage and custom stainless-steel mold, designs of 3D-printed seeding accessories, fluorescent microscopy images of co-culture seeding of inner layer of scaffolds after co-culture seeding using 3D printed accessories, FIJI-ImageJ epithelial coverage code (PDF)
