# Supplementary material for: Method for Targeted Cellular Seeding of Tubular Tissue-Engineered Scaffolds for Tracheal Regeneration Approaches
Source: ACS Biomater Sci Eng. 2025 Aug 7;11(9):5293–305. doi: 10.1021/acsbiomaterials.5c00365 (PMC12421502; doi:10.1021/acsbiomaterials.5c00365)
Supplement: Supplementary file 1 [file ab5c00365_si_001.pdf]

Supporting Information for Publication: Method for Targeted Cellular Seeding of Tubular Tissue-Engineered Scaffolds for Tracheal Regeneration Approaches.

Luis Soriano [1,2,3], Mark Lemoine [2,4], Brenton Cavanagh [5], Anna Johnston[1,2,3], Tehreem Khalid [1,2,4], Fergal J. O'Brien [2,4,6] Cian O'Leary [1,2,3,4,6] and Sally-Ann Cryan [1,2,3,4,6].

[1] School of Pharmacy and Biomolecular Sciences, RCSI University of Medicine and Health Sciences, Dublin, Ireland D02 YN77

[2] Department of Anatomy & Regenerative Medicine, Tissue Engineering Research Group (TERG), RCSI University of Medicine and Health Sciences, Dublin, Ireland D02 YN77

[3] CÚRAM, SFI Research Centre for Medical Devices, University of Galway, Ireland H91 W2TY

[4] SFI Advanced Materials and Bioengineering Research (AMBER) Centre, RCSI University of Medicine and Health Sciences and Trinity College Dublin, Dublin, Ireland D02 YN77, D02 R590

[5] Cellular and Molecular Imaging Core, RCSI, Dublin 2, Ireland D02 YN77

[6] Trinity Centre for Biomedical Engineering, Trinity College Dublin, Dublin, Ireland D02 R590

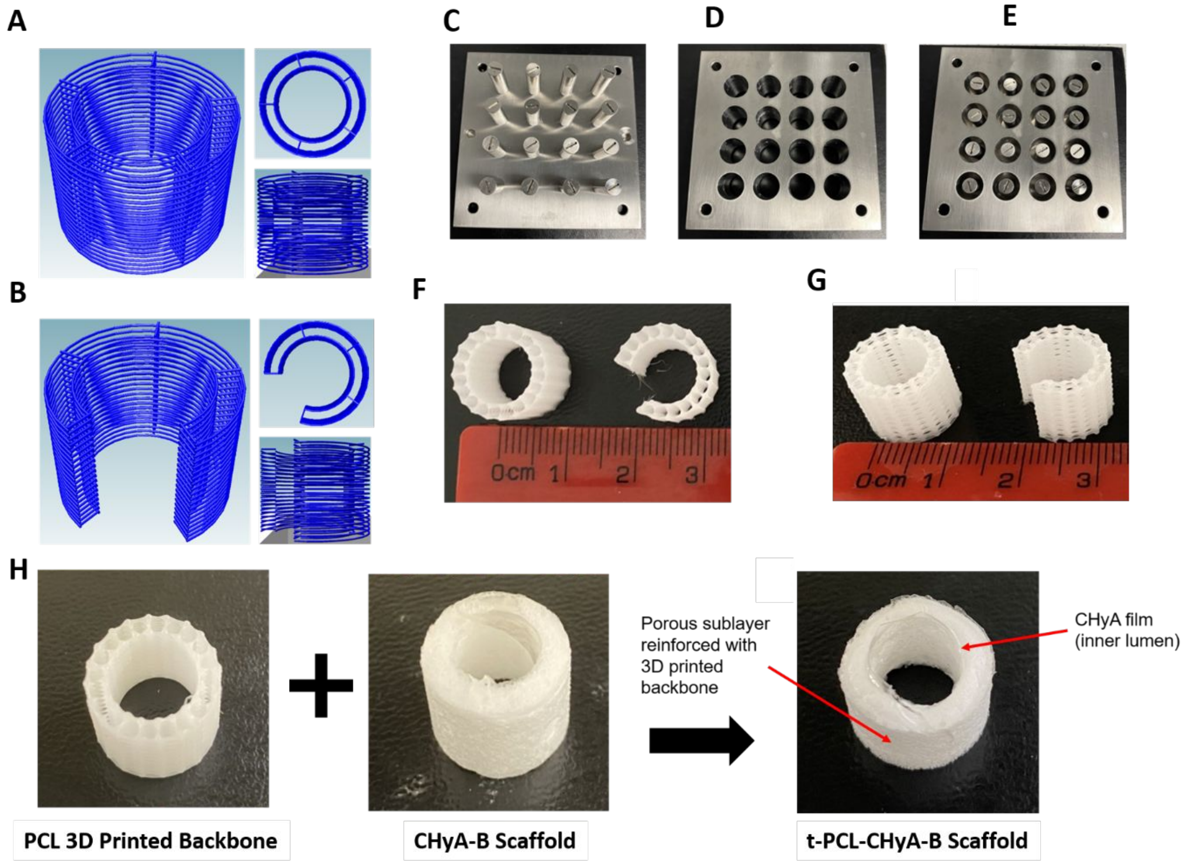

Figure S1. Manufacture of t-PCL-CHyA-B and c-PCL-CHyA-B scaffolds. CAD designs used to generate the 3D printed backbone using PCL and (A) tubular design or (B) c-shape design to mimic the trachea as a hollow tube or the cartilaginous reinforcement. Both designs consisted of 40 layers (0.25 mm each) of 4.8 and 6.6 mm inner and outer radius interconnected with 5 spokes in each layer. Custom stainless-steel mould used to freeze-dry t-PCL-CHyA-B and c-PCL-CHyA-B scaffolds: (A) top plate, (B) bottom plate and (C) mounted mould. 3D printed tubular (F) and c-shape (G) PCL backbone. (H) Combination of 3DP PCL backbone with CHyA-B scaffolds to develop t-PCL-CHyA-B and c-PCL-CHyA-B scaffolds.

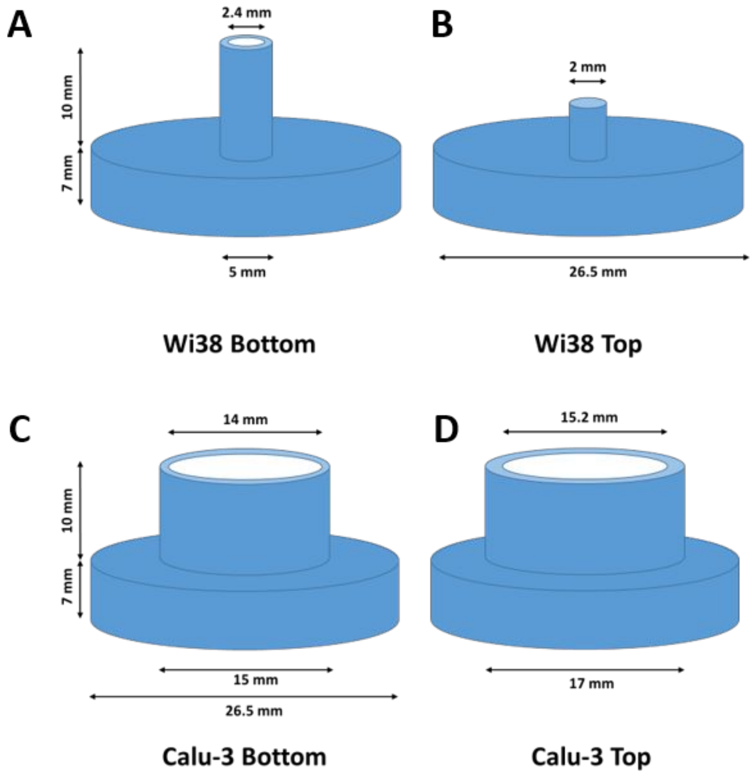

Figure S2. Designs used to 3D print accessories to seed t-PCL-CHyA-B scaffolds. Accessories used for Wi38 seeding (A,B) and Calu-3 seeding (C,D).

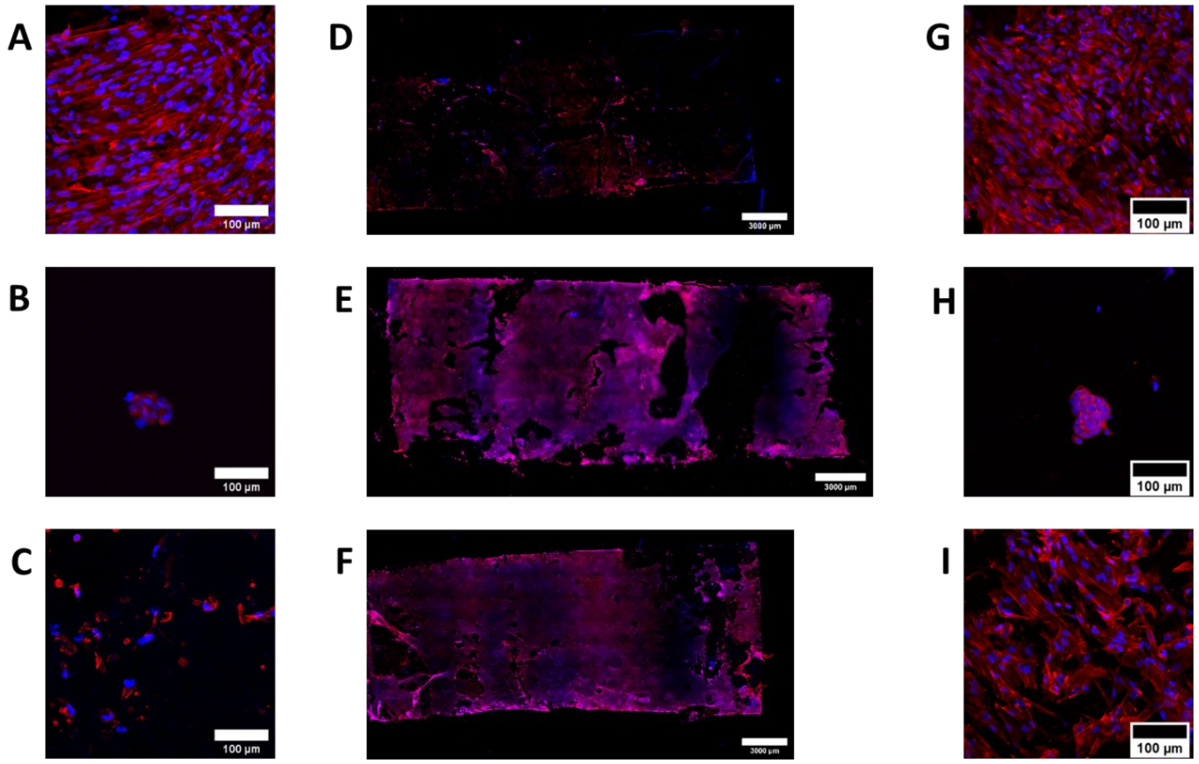

Figure S3. Co-Culture seeding of t-PCL-CHyA-B scaffolds using Wi38 and Calu-3 cells using 3D printed accessories. Representative image of the OL using 4h seeding time: (A) Wi38 monoculture, (B) Calu-3 monoculture and (C) co-culture. Whole film scan of the IL using 2h seeding time: (D) Wi38 monoculture, (E) Calu-3 monoculture and (F) co-culture. Representative image of the OL using 2h seeding time: (G) Wi38 monoculture, (H) Calu-3 monoculture and (I) co-culture (n=3). Cells were stained with DAPI (blue) to indicate the nucleus and phalloidin (red) to identify actin filaments. Imaging was carried out using an Axio Examiner.Z1 confocal microscope.

FIJI-Image J Epithelial Coverage Code

```
/*
 * Script written by Brenton Cavanagh 2021 brentoncavanagh@rcsi.ie
 * Designed to aid user in defining the film area and then calulating
 * the area covered by the cells.
 */

//Gather file information
stack = getTitle();
name = File.nameWithoutExtension;
dir = File.directory;
if(File.exists(dir+File.separator+"results") == 0){
	File.makeDirectory(dir+File.separator+"results");
}
subdir = dir+File.separator+"results";
getDimensions(width, height, channels, slices, frames);

run("Set Measurements...", "area area_fraction limit display redirect=None decimal=4");
name = File.nameWithoutExtension;
savename = subdir+File.separator+name;

//Prepare workspace
run("ROI Manager...");
roiManager("reset");
run("Clear Results");
roiManager("Show All");

//Set threshold and image names
run("Split Channels");
selectWindow("C1-"+stack);
selectWindow("C2-"+stack);
```

```
rename(name+"_Film");
run("8-bit");
setAutoThreshold("Triangle dark");
run("Threshold...");
waitForUser("Check threshold", "Adjust the threhold if needed\n \nPress ok to continue");

//Define film area
run("Analyze Particles...", "size=5000000-Infinity add");
setTool("polygon");
run("ROI Manager...");
run("Brightness/Contrast...");
run("Enhance Contrast", "saturated=0.35");
selectWindow(name+"_Film");
roiManager("Show All");
waitForUser("Check film detection is accurate", "If more than one region is detected,\n \n1. Delete incorrect regions\n \nIf detected regions are correct\n \n1. Delete all regions\n2. Manually outline the film\n3. Add to ROI manager (shortcut key is t)\n \nPress ok to continue");

roiManager("Select", 0);
roiManager("Rename", "Film_area");
roiManager("Set Color", "cyan");
roiManager("Set Line Width", 5);

//Define cells
selectWindow("C1-"+stack);
rename(name);
setAutoThreshold("Default dark");
run("Threshold...");
waitForUser("Check threshold", "Adjust the threhold if needed\n \nPress ok to continue");
run("Create Selection");
roiManager("Add");
roiManager("Select", 1);
```

```
roiManager("Rename", "Cells");
roiManager("Select", newArray(0,1));
roiManager("AND");
roiManager("Add");
roiManager("Select", 2);
roiManager("Rename", "Cell_area");
roiManager("Set Color", "green");
roiManager("Set Line Width", 2);

//measure ROI's
selectWindow(name);
roiManager("Select", newArray(0,2));
run("Set Measurements...", "area area_fraction display redirect=None decimal=4");
roiManager("Measure");

//Save results & ROIs
roiManager("Deselect");
roiManager("Save", savename+".zip");
selectWindow("Results");
saveAs("Text", savename+".csv");

//Save verification image for user
selectWindow(name);
resetThreshold();
roiManager("Show All");
run("Flatten");
saveAs("jpeg", savename);
run("Close All");
```
